# Lessons for the Next Global Health Crisis: A Qualitative Systematic Review of Women's Experiences of the Perinatal Period During the COVID‐19 Pandemic in Australia

**DOI:** 10.1111/ajo.70054

**Published:** 2025-08-06

**Authors:** Ashleigh Shipton, Fanhong Shang, Melissa Wake, Sharon Goldfeld, Fiona Mensah

**Affiliations:** ^1^ Department of Paediatrics University of Melbourne Melbourne Australia; ^2^ General Medicine, Royal Children's Hospital Melbourne Australia; ^3^ Generation Victoria Murdoch Children's Research Institute Melbourne Australia; ^4^ Centre for Community Child Health Royal Children's Hospital Melbourne Australia; ^5^ Population Health Murdoch Children's Research, Institute Melbourne Australia; ^6^ Intergenerational Health Group Murdoch Children's Research Institute Melbourne Australia

**Keywords:** Australia, COVID‐19, health, pregnant women, qualitative systematic review

## Abstract

**Background:**

During the coronavirus disease of 2019 (COVID‐19) pandemic, pregnant women and new mothers in Australia experienced extreme pandemic societal responses but low SARS‐CoV‐2 incidence. This offers one of the few opportunities internationally to learn from the pandemic's indirect effects on maternal health, informing future policy.

**Aims:**

To explore women's qualitative experiences of pregnancy to the 12 postpartum months during the COVID‐19 pandemic in Australia.

**Materials and Methods:**

A systematic search followed PRISMA guidelines. MEDLINE, Embase, Web of Science and PubMed were searched from 1 January 2020, to 13 August 2023, using four categories of terms: ‘COVID‐19’, ‘perinatal’, ‘qualitative’, ‘Australia’. Studies were scored using the CASP checklist and common themes identified from thematic synthesis. The ENTREQ reporting statement was followed.

**Results:**

From eight peer‐reviewed studies, four themes were identified: (1) ‘No one can give you any answers’: Provision of information was inadequate in supporting women to make health‐related decisions; (2) ‘Very isolated’ or ‘It brought us closer’: Social distancing restrictions caused major changes within women's informal support networks; (3) ‘Have they seen enough of me?’: Women felt unsupported during disruptions in maternal health services; (4) ‘All you want to do is keep safe’: Safeguarding family from SARS‐CoV‐2 added cognitive strain to women's daily decision‐making and routine. All studies were of a good or high quality.

**Conclusions:**

Three lessons were highlighted. First, women need accurate, accessible health information to make informed decisions. Second, policies should support family bonding and social connections during government restrictions. Finally, health services must be strengthened to ensure continuous, high‐quality, accessible care during global crises.

## 1 Introduction

On 5 May 2023, the World Health Organization declared the end of the coronavirus disease of 2019 (COVID‐19) pandemic acute phase [1, 2]. The pandemic‐driven changes to health services and policy during this acute phase were so seismic in Australia as to immediately reduce preterm births and increase preterm stillbirths [3, 4]. In anticipation of the inevitable ‘next time’, now presents a rare window of opportunity to learn what shifts in societal, political and economic conditions mattered most to pregnant women and new mothers amidst a global health crisis [5]. Australia is uniquely placed for exploring these indirect effects of the pandemic since there were relatively few SARS‐CoV‐2 cases during long periods of public health restrictions [6, 7].

Notable COVID‐19 policies implemented by the Australian federal and state governments include: the reductions in maternal and child health appointments; restrictions to support persons at antenatal and postnatal appointments; shifts to phone or video appointments (‘telehealth’); closures of early childcare centres; additional provision of welfare subsidies; domestic and international border restrictions; and stay‐at‐home requirements [8, 9], the latter being particularly relevant for those living in Australia's second‐largest city, Melbourne, which became one of the most ‘locked down’ cities in the world [10].

Australia's implementation of public health restrictions was highly effective; not only did it reduce the rates of disease, hospitalisations and deaths early in the pandemic compared to other high‐income countries, but it was also one of only 32 countries to record an increase rather than a decrease in average life expectancy from 2019 to 2021 [11]. However, given the nature of the crisis, untested policy changes precluded consultation with the one million Australian women who gave birth during the 3 years of the acute phase [12]. The voices of these women are particularly important given the heightened need for timely health services and social supports during the perinatal period and the critical influence of pregnancy outcomes in shaping the health of the next generation [13, 14].

Findings from a narrative review and meta‐synthesis regarding women's experiences of previous emergencies suggest that women are vulnerable to social and financial hardship and psychological distress during and following public health emergencies [15, 16]. Building on these learnings, the aim of this systematic review is to synthesise the existing Australian qualitative literature to explore women's experience of pregnancy, birth and the first 12 months postpartum during the COVID‐19 pandemic. A review of this type demands synthesis of qualitative literature, as opposed to quantitative, to ensure an in‐depth, rich analysis of complex environmental changes during the already major life transition of becoming a parent [17].

## 2 Methods and Materials

The review was reported at each stage in accordance with the Enhancing Transparency in Reporting the Synthesis of Qualitative Research (ENTREQ) statement and the Preferred Reporting Items for Systematic Reviews and Meta‐Analyses (PRISMA) statement (Appendices S1 and S2) [18]. A review of this type did not require ethics approval or protocol and was unregistered. A comprehensive search strategy was developed a priori, informed by an expert librarian at the Royal Children's Hospital. The search strategy used MEDLINE, Embase, Web of Science and PubMed databases and reference lists were searched from 1 January 2020 to 13 August 2023. The search included relevant subject heading terms, keywords and word variants within four categories: ‘COVID‐19’, ‘perinatal’, ‘qualitative’ and ‘Australia’ (Appendix S3).

### 2.1 Eligibility Criteria

Inclusion criteria were based on the population, concept and context (Table 1) [19]. There were no language restrictions. Studies were included if a qualitative research design was used. However, designs with insufficient depth of qualitative data to provide rich insights, such as an open‐ended question in an online survey, were excluded. Case reports, comments, editorials, guidelines and letters were also excluded.

**TABLE 1:** Population, Concept and Context Inclusion Criteria.

Population	Women during the perinatal period (from pregnancy to the first 12 months postpartum)
Concept	Lived experiences of the perinatal period during the COVID‐19 pandemic
Context	Located in Australia during the years 2020–2023 (the acute phase of the COVID‐19 pandemic).

### 2.2 Screening

Covidence 2 was used for screening [20]. Two researchers (Author 1, Author 2) independently completed the screening of titles, abstracts and full texts, resolving conflicts by consensus using the eligibility criteria.

### 2.3 Data Extraction

Two reviewers (Author 1, Author 2) independently extracted data for the first three studies to ensure that all necessary data were captured appropriately. Any conflicts were recorded and resolved by consensus using the eligibility criteria. Author 1 then completed data extraction on the remaining studies. The following data were extracted: access date, study identification, journal, publication year, setting, title, first author's name and contact details, life‐course stage (pregnancy, birth, postnatal), study design, aim, period of sampling and data collection, eligibility criteria, method of recruitment, sample size, baseline characteristics, themes and participant quotations.

### 2.4 Critical Appraisal

Each study was scored according to the 2019 Critical Appraisal Skills Program (CASP) qualitative research checklist as recommended by Cochrane [17, 21, 22]. The checklist is designed to systematically assess rigour using a set of 10 questions (Table 2). Study quality was rated below acceptable, acceptable, good, or high quality if < 6, 6, 7–9, or all 10 of the items respectively were answered ‘yes’ [23]. Two reviewers (Author 1, Author 2) independently extracted data for the first three studies to ensure that all necessary data were captured appropriately. Any conflicts were recorded and resolved by consensus, referring back to the CASP criteria. Author 1 then completed data extraction on the remaining studies.

**TABLE 2:** Study characteristics.

Author (year)	Study setting	Study period	Inclusion criteria	Method of recruitment	Number of participants	Method of data collection	Method of data analysis	Lifecourse stage	Topic of research question
Atchan (2023)	Australia (states and territories not specified), New Zealand	September–October 2020	Women (not limited to those breastfeeding) who had a baby in Australia or New Zealand during the preceding months of the COVID‐19 pandemic, since March 2020.	Social media advertising, targeted to early parenting social media groups, through Facebook, Twitter and other online platforms.	27	Semi‐structured interviews	Thematic analysis	Postnatal	Breastfeeding
Atmuri (2021)	Victoria	June 2020	Women pregnant at any gestation at the time of recruitment who were receiving antenatal care at the study hospital.	Face‐to‐face at an antenatal outpatient clinic.	15	Semi‐structured interviews	Thematic analysis	Pregnancy	Perspectives on pregnancy
Caddy (2023)	Western Australia, Victoria, Northern Territory, Queensland, Australian Capital Territory, South Australia, New South Wales, Tasmania	June–July 2021	Women pregnant since March 2020, Australian residents, aged at least 18 years, proficient in English and with access to a computer or phone for interview.	Online social media advertising.	21	Semi‐structured interviews	Thematic analysis	Perinatal	Accessing information
Davis (2021)	Western Australia	November 2020–February 2021	Women who were eligible for the ORIGINS project sub study, ‘the Community Wellbeing Project’, who were expecting a baby in 2020 and had completed online questionnaires on their experience of living through the COVID‐19 pandemic.	Existing participants of ORIGINS project sub study, ‘The Community Wellbeing Project’, were provided with information about the study via text message.	14	Semi‐structured interviews	Thematic analysis	Perinatal	Emotional health and wellbeing
Hood (2021)	Western Australia	July–September 2020	Women who were eligible for the ORIGINS project who had an infant aged 9 to 15 months at the time of the interview, were proficient in English and were available for an interview by audio call or video call.	Existing participants of ORIGINS project were provided with information about the study via a mobile touch screen device and recruited via email and phone call.	30	Semi‐structured interviews	Thematic analysis	Postnatal	Family routine, relationship and technology use
Oliver (2022)	Victoria	July–October 2021	Women who were eligible for The Optimise Study, were aged 18–40 years and lived in Victoria.	Existing participants of The Optimise Study cohort were contacted as well as Facebook advertisement targeting pregnant/breastfeeding women who were unsure or not intending to accept a COVID‐19 vaccine.	24	Semi‐structured interviews	Thematic analysis	Perinatal	COVID‐19 vaccine hesitancy
Sweet (2021)	Western Australia, Victoria, Northern Territory, Queensland, Australian Capital Territory, South Australia, New South Wales, Tasmania	June 2020	Women who had completed the online survey in phase one of the Sweet (2021) study. Eligibility criteria for phase one included those who had received maternity care, were pregnant or had given birth since March 2020.	Social media advertising on Facebook, Twitter and Instagram and researchers' professional networks. The Australian College of Midwives and the Royal Australian and New Zealand College of Obstetricians and gynaecologists also advertised the study through their online member communication systems.	27	Semi‐structured interviews	Thematic analysis	Perinatal	Childbearing experience
Zinga (2022)	Victoria	August–November 2020	Women who were English‐speaking, adult and pregnant attending antenatal clinics at study hospital and were experiencing financial hardship and/or food insecurity.	Advertising flyers at study hospital antenatal clinics and posts on study hospital social media platforms.	7	Semi‐structured interviews	Thematic analysis	Pregnancy	Food insecurity

### 2.5 Thematic Synthesis

Thematic synthesis was the chosen approach for synthesising data as endorsed by the 2023 Cochrane‐Campbell Handbook for Qualitative Evidence Synthesis [24, 25, 26]. This process was completed through the consensus of two reviewers (Author 1, Author 2).

In the first phase, full texts were imported into NVivo 20 and reviewers Author 1 and Author 2 independently familiarised themselves with the data through several rounds of reading and noting initial observations [27, 28]. Each study's data relevant to this review's aim were then systematically synthesised by Author 1 in close consultation with Author 2 [27]. The data synthesised were all the direct quotations from participants (Appendix S4) and author interpretations of the included studies. As informed by Braun and Clarke (2022), data were firstly synthesised into codes, with related codes then merged into a smaller number of subthemes and related subthemes then merged into a smaller number of final themes [27]. A primarily inductive approach was taken, so that codes remained close to the study authors' interpretations, thus avoiding going beyond what studies were reporting [25, 27]. This process was recursive and iterative, involving several cycles of reviewers Author 1 and Author 2 collaboratively revisiting and refining results [27]. Each stage was documented so that an audit trail of decisions would enable the ability to trace conclusions directly back to the study on which the synthesis was based [26].

Only codes and themes that appeared in more than one study were included in the final results [29]. The common themes were then displayed in a narrative format of descriptive paragraphs with a selection of embedded quotations and also formatted into a table with corresponding quotations (Appendix S4).

## 3 Results

### 3.1 Study Characteristics

**FIGURE 1:**
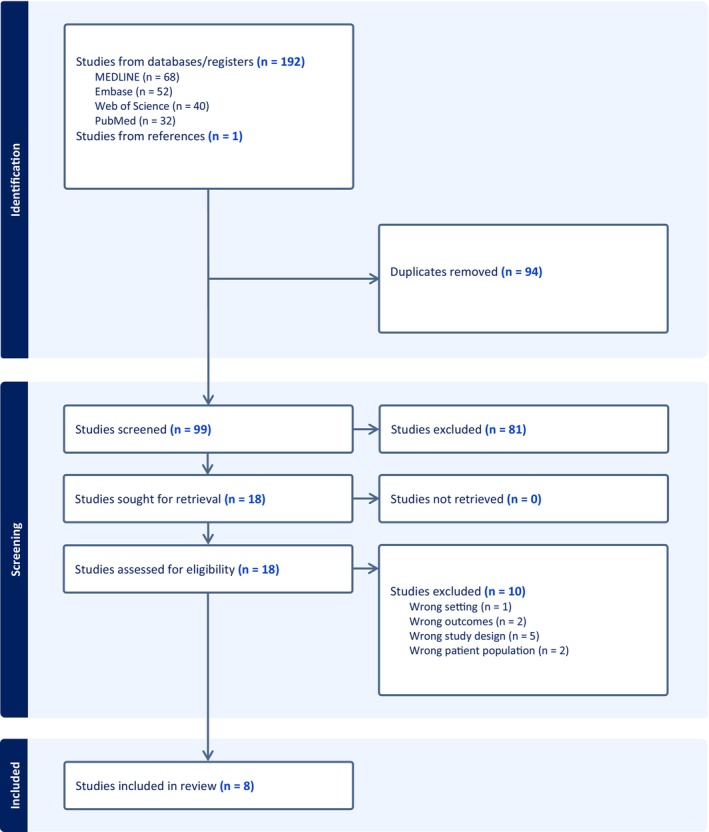
Results from a systematic search following PRIMSA guidelines.

From 193 studies, eight were included [30, 31, 32, 33, 34, 35, 36, 37] (Figure 1). Each study had a unique research focus: accessing information; breastfeeding; emotional health and wellbeing; family routine, relationship and technology; food insecurity; childbearing experiences; COVID‐19 vaccine hesitancy; and perspectives on pregnancy, respectively (Table 2). Five studies collected data from 2020, two from 2021, and one from both 2020 and 2021. Three studies focused on pregnancy, two on the postnatal period, and four on the whole perinatal period. All studies conducted semi‐structured interviews using thematic analysis. None of the studies was excluded based on rigour since three studies were of high quality and five were of good quality (Table 3).

**TABLE 3:** CASP checklist results.

Author (year)	CASP appraisal question										Quality
	Q1	Q2	Q3	Q4	Q5	Q6	Q7	Q8	Q9	Q10	Overall
Atchan (2023)	Y	Y	Y	Y	Y	Y	Y	Y	Y	Y	High
Atmuri (2022)	Y	Y	Y	Y	Y	Y	Y	Y	Y	Y	High
Caddy (2023)	Y	Y	Y	Y	Y	N	Y	Y	Y	Y	Good
Davis (2021)	Y	Y	Y	Y	Y	N	Y	Y	Y	Y	Good
Hood (2021)	Y	Y	Y	Y	Y	N	Y	Y	Y	Y	Good
Oliver (2022)	Y	Y	Y	Y	Y	N	Y	Y	Y	Y	Good
Sweet (2021)	Y	Y	Y	Y	Y	N	Y	Y	Y	Y	Good
Zinga (2022)	Y	Y	Y	Y	Y	Y	Y	Y	Y	Y	High

*Notes:* Questions 1–10: Q1. Was there a clear statement of the aims of the research? Q2. Is a qualitative methodology appropriate? Q3. Was the research design appropriate to address the aims of the research? Q4. Was the recruitment strategy appropriate to the aims of the research? Q5. Was the data collected in a way that addressed the research issue? Q6. Has the relationship between researcher and participants been adequately considered? Q7. Have ethical issues been taken into consideration? Q8. Was the data analysis sufficiently rigorous? Q9. Is there a clear statement of findings? Q10. Was the study valuable (considering if the authors discuss the contribution the study makes to existing knowledge or understanding, if they identify new areas where research is necessary and if they have discussed whether or how the findings can be transferred to other populations or considered other ways the research may be used)?

*Abbreviations:* CASP, Critical Appraisal Skills Programme; N, no; U, unsure/can't tell; Y, yes.

### 3.2 Common Themes

Appendix S4 provides the comprehensive list of participant quotations synthesised into the themes listed in Table 4 and explored below.

**TABLE 4:** Summary of common themes and corresponding studies.

Theme	Studies reporting on theme
‘No one can give you any answers’: Provision of information was inadequate in supporting women to make health‐related decisions.	Atchan 2023, Atmuri 2022, Caddy 2023, Davis 2021, Oliver 2022, Sweet 2021
‘Very isolated’ or ‘It brought us closer’: Social distancing restrictions caused major changes within women's informal support networks.	Atchan 2023, Atmuri 2022, Caddy 2023, Davis 2021, Sweet 2021, Hood 2021
‘Have they seen enough of me?’: Women felt unsupported during disruptions in maternal health services.	Atchan 2023, Atmuri 2022, Caddy 2023, Davis 2021, Sweet 2021
‘All you want to do is keep safe’: Safeguarding family from SARS‐CoV‐2 added cognitive strain to women's daily decision making and routines.	Atchan 2023, Atmuri 2022, Caddy 2023, Hood 2021, Sweet 2021, Zinga 2022

Theme 1 *‘No one can give you any answers’: Provision of information was inadequate in supporting women to make informed health‐related decisions*.

This theme explores women's experiences of health information provision during the pandemic and their responses to any barriers encountered. Health information sought by women commonly included routine perinatal care guidance, COVID‐19 policy updates, the effect of SARS‐CoV‐2 on pregnant women and infants, and the safety of COVID‐19 vaccinations. Six studies contributed to this theme (see Appendix S4 for additional quotations) [30, 31, 32, 35, 36, 37].

In general, women reported poor experiences of the communication of health information from health services and government agencies [30, 31, 32, 35, 36, 37]. ‘When they cancelled everything, it took weeks for them to do anything online … you try to call to find out some answers and no one can give you any answers and there was no one to talk to.’ [32] Information was reportedly outdated, contradictory, insufficient, difficult to understand, hard to find and delayed [30, 31, 32, 35, 36, 37]. “We [pregnant women] were being told ‘that’ and now we're being told ‘this’. What if we get told something in a few months' time that contradicts the action we've taken [getting vaccinated]? …” [36].

As a result, women were left feeling anxious, stressed and frustrated [30, 32, 36, 37]. Some felt uninformed for parenthood and unsupported to make health decisions or adopt risk reduction strategies such as getting the COVID‐19 vaccinations [30, 36, 37]. In response to missed health information, women proactively did their own research [30, 31, 32, 35, 36, 37]. Many turned to online resources, such as Google, social media or government websites [30, 31, 35, 36, 37] ‘ … anytime the rules changed, you know, within a couple of hours, they would have posted on Instagram …’ [30]. With disinformation online and a lack of trust in mainstream media, some women sought information from social media accounts managed by trustworthy health providers, hospitals or scientific organisations [30, 35, 36].

Theme 2 *‘Very isolated’ or ‘It brought us closer’: Social distancing restrictions caused major changes within women's informal support networks*.

This theme captures women's experience of shifts in social networks of family, friends and other new parents. Six studies contributed to this theme (see Appendix S4 for additional quotations) [30, 31, 32, 33, 35, 37].

A positive outcome from the mandatory stay‐at‐home requirements was the enhancement of family bonding within the immediate household [31, 32, 33, 35]. The slowing down of daily life and the reduced pressure to socialise created opportunities for uninterrupted time to bond with their newborn, particularly helpful for women establishing breastfeeding [31]. “I just think it's been almost a blessing in disguise…” [31]. Flexible work arrangements also meant that some families benefited from both parents being at home [32, 33]. “Since this COVID‐19 started my husband is at home more, so that's when I see that he's [the child is] getting more closer to his dad.” [33]. Daily routines shifted to mostly home‐based family activities [33].

The mandatory stay‐at‐home requirements also contributed to women experiencing loneliness and isolation [31, 32, 33, 35, 37] ‘It's been very impersonal, being pregnant for the first time, I find myself not having anyone to talk to and it does feel very alone…’ [35] Some spoke of ‘missing out’ on the traditional pregnancy rituals and the valued involvement of their friends and family in their pregnancy and care of their newborn [32, 33, 35, 37]. Women with older children expressed concern for first‐time mothers learning new skills, such as breastfeeding, without the usual supports [31, 35]. ‘I think if I had been a first‐time mum it would have been a lot harder…not knowing what services were around – how to access them and how they were being delivered differently that would have been a huge barrier.’ [35]. Additionally, the activities where new parents would usually connect and exchange advice, such as antenatal groups, ‘rhyme time’, ‘baby sensory’ or ‘Gymbaroo’, were cancelled [32, 33, 37].

Technology was a vital resource used to connect with friends, family and other parents outside of the immediate household, particularly through phone calls, videoconferencing, messaging and joining online groups [30, 32, 33, 35]. Connection with other parents was often enabled through social media groups, providing both support and a sense of comfort knowing that others were going through a similar experience of parenthood during a pandemic [30, 32, 35]. Some women also joined online activities such as exercise classes and baby sensory classes to find connection and maintain a sense of normality [33].

Theme 3 *‘Have they seen enough of me?’: Women felt unsupported during disruptions in maternal health services*.

This theme highlights women's experience of major changes to perinatal and mental health services. Five studies contributed to this theme (see Appendix S4 for additional quotations) [30, 31, 32, 35, 37].

Overall, women experienced rapid transformations to health services [30, 31, 32, 35, 37]. They encountered several challenges including barriers to access, delays in receiving support from a health professional, frequent appointment cancellations and shortened appointment durations [30, 31, 32]. The shift to telehealth was experienced by many as impersonal and compromising on quality with a common concern being the inability of health providers to physically examine or weigh infants [31, 32, 37]. ‘ … lactation needs to be face‐to‐face – like the Zoom was quite tricky … and by the time we got to face‐to‐face it was almost too late.’ [31]. Additional stressors were the inability to have a support person at appointments or during labour and the potential for early discharge from hospital [32, 35, 37].

These rapid transformations in health services left women feeling uncertain, unsupported and alone [32, 35, 37]. It also contributed to anxiety, stress and low mood [31, 32, 35, 37]. Some women felt that these changes delayed the diagnosis of common infant and maternal conditions, such as faltering growth or mastitis, and negatively impacted their ability to establish breastfeeding [31].

Theme 4 *‘All you want to do is keep safe’: Safeguarding family from SARS‐CoV‐2 added cognitive strain to women's daily decision making and routines*.

This theme explores how women's daily routine and decision‐making were influenced by feeling as though they had to protect themselves and family against the SARS‐CoV‐2 virus. Seven studies contributed to this theme (see Appendix S4 for additional quotations) [30, 31, 33, 34, 35, 36, 37].

Despite an initial low prevalence of SARS‐CoV‐2 in Australia, keeping safe from the virus still heavily shaped women's daily decision making and routine [30, 33, 34, 35, 37]. ‘There's a lot of fear. What happens if I do get COVID? Is that gonna affect my pregnancy? … how's that gonna affect the baby potentially?’ [30]. Some feared that if they or their partner became infected with SARS‐CoV‐2, they would have to birth alone or be separated from their newborn [30, 37].

Deciding whether or not to have the COVID‐19 vaccination was an additional cognitive strain on pregnant women and new mothers [36]. Some women's decision to have the vaccination was based on the transferred immunity properties protective for their infant [36]. A similar rationale was used by some women who wanted to exclusively breastfeed their infant or delay weaning for the transferred immunity benefits against SARS‐CoV‐2 [31].

A few women adapted their grocery shopping routine by shopping online or going early in the morning to avoid interaction with the public [33, 34, 35]. Others kept their child out of daycare or cancelled childcare from elderly relatives [33, 35]. Some women even ceased employment [37]. “I actually stopped going to work … I'm a kindergarten teacher and it was just recommended that given the limited research on what would happen.” [37]. Even when restrictions were lifted, some women continued to avoid public spaces [33]. “We haven't re‐joined any of our classes that we did before … we're still a bit wary when we go out.” [33]. The uncertainty and fear of SARS‐CoV‐2 ultimately led to stress and anxiety [30, 33, 35, 37].

## 4 Conclusions

The four main themes identified in this qualitative systematic review organise a powerful set of insights into women's experiences of the perinatal period during the COVID‐19 pandemic in Australia. These themes are consistent with previous international qualitative reviews and meta‐syntheses which also report that women in other countries experienced poor information provision, changes to social supports and family dynamics, barriers in accessing maternal healthcare, and the adoption of infection control behaviours [23, 29, 38, 39]. The consistency among international literature suggests some shared experiences in becoming new parents during the pandemic despite global variations in policy and SARS‐CoV‐2 prevalence.

Together, *Themes 1–4* highlight a series of simple, urgent and cogent lessons relevant to policy and health service planning for the inevitable ‘next time’. A key learning from *Theme 1* is that pregnant women and new mothers need accurate and accessible health information to make informed decisions. Health providers and government agencies play vital roles in communicating credible information during emerging health risks [40]. A 2022 Australian review into communication during the pandemic recommended that governments should also use local organisations and trusted individuals to deliver public health messages, counteract misinformation and reach specific cohorts, such as pregnant women and new mothers [41]. Women in our review emphasised this by noting the important presence of a trusted health professional on social media to provide health information. Beyond the pandemic, health providers and governments should maintain an active presence on women's preferred communication channels, intentionally building their trust and combatting misinformation [42]. With the rise in widely accessible Artificial Intelligence alongside minimal guardrails protecting information accuracy, this will become increasingly important [42].

An implication from *Theme 2* is how policies could support family bonding and social connections during government restrictions. Social connection is a protective factor against perinatal mental illness [43]. This is particularly relevant given (a) how common perinatal mental illness is, with one in five women in Australia experiencing perinatal depression and anxiety, (b) its substantial economic toll, with the Australian government spending over half a billion dollars each year and (c) the stress and anxiety experienced by women during the pandemic as highlighted in *Themes 1–4* [44].

Online groups were an important initiative for combatting social isolation during government restrictions, as suggested by our review. Future investment into digital solutions should consider that women are one of the most at‐risk groups for digital exclusion in Australia, meaning they are at risk of being left behind and facing barriers when interacting with a digitised society [45, 46]. Without waiting for the next pandemic to address this issue, initiatives such as Good Things Foundation Australia are already helping to bridge this digital divide, ensuring equitable access to digital solutions [46].

Policymakers could also look into continuing the benefits of enhanced family bonding during stay‐at‐home requirements by adopting comparable policies going forward. Paid parental leave is one such policy facilitating more time at home after childbirth. In Australia, paid parental leave leads to lengthened breastfeeding durations, reduced maternal stress and decreased relationship tensions, and a secure infant attachment enhances infant brain development [14, 47]. The Australian Senate recently passed legislation to extend the current 20 weeks per family of paid leave to 26 weeks, beginning 2026 [48]. Whilst this policy is below the Organisation for Economic Co‐operation and Development average of 39 weeks paid parental leave, it represents positive post‐pandemic progress [49].

A learning from *Theme 3* is that maternal health services need to be better resourced to ensure continuous, high‐quality and accessible care during global crises. Women's experiences exposed the vulnerabilities in Australia's maternal health services. Understandably, these services were placed under significant pressure during the pandemic [8, 50]. However, key consequences highlighted by women in this review were delays to care (a known risk factor for adverse maternal and infant health outcomes) and not feeling reassured by telehealth consultations in the setting of issues requiring physical examinations [13].

Future‐proofing maternal health services for the next global health crisis may involve (a) recognising that women in the perinatal period are a high‐risk population requiring the continuation of timely, high‐quality, accessible care to prevent adverse outcomes, (b) prioritising additional resources during a crisis such as mental health support given the heightened stress highlighted in *Theme 4* and (c) building a health service that focuses on the prevention of disease to create the healthiest possible population at baseline before the next health crisis [5, 51]. Additionally, telehealth may play an adjunct role in post‐pandemic maternity services, but is not a suitable replacement for face‐to‐face care according to a 2024 Australian study [52]. Looking ahead, telehealth could alongside the incorporation of promising photogrammetry technology could be further researched in the effectivness of screnning for conditions that currently require physical examinations [51, 53].

This is the first qualitative systematic review to thematically synthesise existing literature of Australian women's experiences of the perinatal period during the COVID‐19 pandemic. All included studies were of good or high rigour and used interviews to collect in‐depth data. Although eight studies were included, not each study contributed to every theme.

A key limitation is the convenience sampling of participants used in some studies from pre‐existing longitudinal studies or online platforms. This is understandable given the recruitment constraints during the pandemic; however, it has compromised the diversity of participant demographics such that higher socioeconomic status and proficiency in English are overrepresented. Future studies should focus on demographic diversity, not for generalisability purposes, but rather to highlight the range of women's experiences. A further limitation of the review is that only common codes or themes were included. By doing so, the authors were not discounting other codes or themes as unimportant; rather, this was a pragmatic method for analysing a large number of codes and themes within the existing literature.

In anticipation of the inevitable next global health crisis, we synthesise here Australian women's experiences of pregnancy, birth and postpartum in the time of COVID‐19. Overall, the basic tenets of trust in public health messaging and a robust maternal health service need to be built long before the onset of future global health crises. The role of digital solutions for accurate information provision, social connection and health services is also central to pandemic preparedness. These lessons provided are simple, urgent and cogent, guiding future policy and health service provision beyond the COVID‐19 pandemic.

## Ethics Statement

The authors have nothing to report.

## Conflicts of Interest

The authors declare no conflicts of interest.

## Supporting information


Appendix S1
