# Supplementary material for: Lessons for the Next Global Health Crisis: A Qualitative Systematic Review of Women's Experiences of the Perinatal Period During the COVID‐19 Pandemic in Australia
Source: Aust N Z J Obstet Gynaecol. 2025 Aug 6;66(1):e70054. doi: 10.1111/ajo.70054 (PMC12867550; doi:10.1111/ajo.70054)
Supplement: Supplementary file 1 — Appendix S1 [file AJO-66-0-s001.pdf]

SUPPORTING INFORMATION

Appendix 1: PRISMA Checklist

Section and Topic	Item #	Checklist item	Location where item is reported
**TITLE**			
Title	1	Identify the report as a systematic review.	1
**ABSTRACT**			
Abstract	2	See the PRISMA 2020 for Abstracts checklist.	1
**INTRODUCTION**			
Rationale	3	Describe the rationale for the review in the context of existing knowledge.	2
Objectives	4	Provide an explicit statement of the objective(s) or question(s) the review addresses.	2
**METHODS**			
Eligibility criteria	5	Specify the inclusion and exclusion criteria for the review and how studies were grouped for the syntheses.	2, Table 1
Information sources	6	Specify all databases, registers, websites, organisations, reference lists and other sources searched or consulted to identify studies. Specify the date when each source was last searched or consulted.	2
Search strategy	7	Present the full search strategies for all databases, registers and websites, including any filters and limits used.	Appendix 3
Selection process	8	Specify the methods used to decide whether a study met the inclusion criteria of the review, including how many reviewers screened each record and each report retrieved, whether they worked independently, and if applicable, details of automation tools used in the process.	2
Data collection process	9	Specify the methods used to collect data from reports, including how many reviewers collected data from each report, whether they worked independently, any processes for obtaining or confirming data from study investigators, and if applicable, details of automation tools used in the process.	2
Data items	10a	List and define all outcomes for which data were sought. Specify whether all results that were compatible with each outcome domain in each study were sought (e.g. for all measures, time points, analyses), and if not, the methods used to decide which results to collect.	2
	10b	List and define all other variables for which data were sought (e.g. participant and intervention characteristics, funding sources). Describe any assumptions made about any missing or unclear information.	2, Table 2
Study risk of bias assessment	11	Specify the methods used to assess risk of bias in the included studies, including details of the tool(s) used, how many reviewers assessed each study and whether they worked independently, and if applicable, details of automation tools used in the process.	2,3
Effect measures	12	Specify for each outcome the effect measure(s) (e.g. risk ratio, mean difference) used in the synthesis or presentation of results.	N/A
Synthesis methods	13a	Describe the processes used to decide which studies were eligible for each synthesis (e.g. tabulating the study intervention characteristics and comparing against the planned groups for each synthesis (item #5)).	3

Section and Topic	Item #	Checklist item	Location where item is reported
	13b	Describe any methods required to prepare the data for presentation or synthesis, such as handling of missing summary statistics, or data conversions.	3
	13c	Describe any methods used to tabulate or visually display results of individual studies and syntheses.	3
	13d	Describe any methods used to synthesize results and provide a rationale for the choice(s). If meta-analysis was performed, describe the model(s), method(s) to identify the presence and extent of statistical heterogeneity, and software package(s) used.	3
	13e	Describe any methods used to explore possible causes of heterogeneity among study results (e.g. subgroup analysis, meta-regression).	N/A
	13f	Describe any sensitivity analyses conducted to assess robustness of the synthesized results.	N/A
Reporting bias assessment	14	Describe any methods used to assess risk of bias due to missing results in a synthesis (arising from reporting biases).	N/A
Certainty assessment	15	Describe any methods used to assess certainty (or confidence) in the body of evidence for an outcome.	3
**RESULTS**			
Study selection	16a	Describe the results of the search and selection process, from the number of records identified in the search to the number of studies included in the review, ideally using a flow diagram.	Figure 1
	16b	Cite studies that might appear to meet the inclusion criteria, but which were excluded, and explain why they were excluded.	Figure 1
Study characteristics	17	Cite each included study and present its characteristics.	3, Table 2
Risk of bias in studies	18	Present assessments of risk of bias for each included study.	3, Table 3
Results of individual studies	19	For all outcomes, present, for each study: (a) summary statistics for each group (where appropriate) and (b) an effect estimate and its precision (e.g. confidence/credible interval), ideally using structured tables or plots.	N/A
Results of syntheses	20a	For each synthesis, briefly summarise the characteristics and risk of bias among contributing studies.	3, Table 2, Table 3
	20b	Present results of all statistical syntheses conducted. If meta-analysis was done, present for each the summary estimate and its precision (e.g. confidence/credible interval) and measures of statistical heterogeneity. If comparing groups, describe the direction of the effect.	3-9, Table 4, Appendix 4
	20c	Present results of all investigations of possible causes of heterogeneity among study results.	N/A
	20d	Present results of all sensitivity analyses conducted to assess the robustness of the synthesized results.	N/A
Reporting biases	21	Present assessments of risk of bias due to missing results (arising from reporting biases) for each synthesis assessed.	N/A
Certainty of evidence	22	Present assessments of certainty (or confidence) in the body of evidence for each outcome assessed.	Table 3
**DISCUSSION**			

Section and Topic	Item #	Checklist item	Location where item is reported
Discussion	23a	Provide a general interpretation of the results in the context of other evidence.	9-10
	23b	Discuss any limitations of the evidence included in the review.	10
	23c	Discuss any limitations of the review processes used.	10
	23d	Discuss implications of the results for practice, policy, and future research.	9-10
**OTHER INFORMATION**			
Registration and protocol	24a	Provide registration information for the review, including register name and registration number, or state that the review was not registered.	2
	24b	Indicate where the review protocol can be accessed, or state that a protocol was not prepared.	2
	24c	Describe and explain any amendments to information provided at registration or in the protocol.	N/A
Support	25	Describe sources of financial or non-financial support for the review, and the role of the funders or sponsors in the review.	1, 10
Competing interests	26	Declare any competing interests of review authors.	10
Availability of data, code and other materials	27	Report which of the following are publicly available and where they can be found: template data collection forms; data extracted from included studies; data used for all analyses; analytic code; any other materials used in the review.	Appendix 4

Appendix 2: ENTREQ Checklist

No	Item	Guide and description	Page(s) Found
**1**	Aim	State the research question the synthesis addresses.	2
**2**	Synthesis methodology	Identify the synthesis methodology or theoretical framework which underpins the synthesis, and describe the rationale for choice of methodology *(e.g. meta-ethnography, thematic synthesis, critical interpretive synthesis, grounded theory synthesis, realist synthesis, meta-aggregation, meta-study, framework synthesis).*	2, 3
**3**	Approach to searching	Indicate whether the search was pre-planned (*comprehensive search strategies to seek all available studies)* or iterative (*to seek all available concepts until they theoretical saturation is achieved)*.	2
**4**	Inclusion criteria	Specify the inclusion/exclusion criteria *(e.g. in terms of population, language, year limits, type of publication, study type).*	2, Table 1
**5**	Data sources	Describe the information sources used (e.g. *electronic databases (MEDLINE, EMBASE, CINAHL, psycINFO, Econlit), grey literature databases (digital thesis, policy reports), relevant organisational websites, experts, information specialists, generic web searches (Google Scholar) hand searching, reference lists)* and when the searches conducted; provide the rationale for using the data sources.	2
**6**	Electronic Search strategy	Describe the literature search *(e.g. provide electronic search strategies with population terms, clinical or health topic terms, experiential or social phenomena related terms, filters for qualitative research, and search limits)*.	2, Appendix 3
**7**	Study screening methods	Describe the process of study screening and sifting *(e.g. title, abstract and full text review, number of independent reviewers who screened studies).*	2
**8**	Study characteristics	Present the characteristics of the included studies *(e.g. year of publication, country, population, number of participants, data collection, methodology, analysis, research questions).*	3, Table 2

No	Item	Guide and description	Page(s) Found
**9**	Study selection results	Identify the number of studies screened and provide reasons for study exclusion *(e,g, for comprehensive searching, provide numbers of studies screened and reasons for exclusion indicated in a figure/flowchart; for iterative searching describe reasons for study exclusion and inclusion based on modifications t the research question and/or contribution to theory development).*	3, Figure 1
**10**	Rationale for appraisal	Describe the rationale and approach used to appraise the included studies or selected findings *(e.g. assessment of conduct (validity and robustness), assessment of reporting (transparency), assessment of content and utility of the findings).*	2, 3
**11**	Appraisal items	State the tools, frameworks and criteria used to appraise the studies or selected findings *(e.g. Existing tools: CASP, QARI, COREQ, Mays and Pope* [25]*; reviewer developed tools; describe the domains assessed: research team, study design, data analysis and interpretations, reporting).*	2, 3, Table 3
**12**	Appraisal process	Indicate whether the appraisal was conducted independently by more than one reviewer and if consensus was required.	2, 3
**13**	Appraisal results	Present results of the quality assessment and indicate which articles, if any, were weighted/excluded based on the assessment and give the rationale.	8, Table 3
**14**	Data extraction	Indicate which sections of the primary studies were analysed and how were the data extracted from the primary studies? *(e.g. all text under the headings "results /conclusions" were extracted electronically and entered into a computer software).*	3
**15**	Software	State the computer software used, if any.	3
**16**	Number of reviewers	Identify who was involved in coding and analysis.	3
**17**	Coding	Describe the process for coding of data *(e.g. line by line coding to search for concepts).*	3
**18**	Study comparison	Describe how were comparisons made within and across studies *(e.g. subsequent studies were coded into pre-existing concepts, and new concepts were created when deemed necessary).*	3

No	Item	Guide and description	Page(s) Found
**19**	Derivation of themes	Explain whether the process of deriving the themes or constructs was inductive or deductive.	3
**20**	Quotations	Provide quotations from the primary studies to illustrate themes/constructs, and identify whether the quotations were participant quotations of the author's interpretation.	3, 7-9, Appendix 4
**21**	Synthesis output	Present rich, compelling and useful results that go beyond a summary of the primary studies (e.g. *new interpretation, models of evidence, conceptual models, analytical framework, development of a new theory or construct).*	3-9, Table 4

Appendix 3: Search terms

Database	COVID-19	Perinatal	Qualitative	Australia
**Ovid MEDLINE**	*COVID-19/ or *SARS-CoV-2/ OR<br>(2019-novel or 2019nCoV or 2019-nCoV or COVID-19 or COVID19 or COVID-2019 or COVID2019 or CONVID-19 or CONVID19 or CORVID-19 or CORVID19 or CoV2 or CoV-2 or HCoV* or Ncov* or Ncorona* or Ncorono* or NcovChina* or NcovChinese* or NcovHubei* or NcovWuhan* or SARS2 or SARS-2 or SARScoronavirus2 or SARScoronavirus-2 or SARScoronovirus2 or SARScoronovirus-2 or SARSCov19 or SARSCov-19 or SARS-CoV-2 or SARSCoV-2 or SARSCoV2 or WN-CoV or WNCoV or wuhan-virus).tw,kf,ot. OR<br>((pneumonia* or outbreak* or respiratory-illness* or respiratory-disease* or respiratory-symptom* or seafood-market* or food-market* or wildlife) and (Wuhan or China or Chinese or Hubei or Huanan)).tw,kf,ot. OR<br>((new or novel or nouveau or "19" or "2019" or Wuhan or Hubei or Huanan or China or Chinese) adj3 (coronavirus* or corona virus* or betacoronavirus* or CoV or HCoV)).tw,kf,ot. OR<br>(longCOVID* or postCOVID* or postcoronavirus* or postSARS*).ti,ab,kf,ot. OR<br>*POST-ACUTE COVID-19 SYNDROME/ or (longCOVID* or postCOVID* or postcoronavirus* or postSARS*).tw,kf,ot.<br>(*coronavirus/ or *betacoronavirus/ or *coronavirus infections/) and (*disease outbreaks/ or *epidemics/ or *pandemics/) OR<br>((coronavirus* or corona-virus* or betacoronavirus*) adj3 (pandemic* or epidemic* or outbreak* or crisis)).tw,kf,ot	Exp fetus/ or exp infant/ or (fetal or foetal or fetus or foetus or preterm or premature or infan* or neonat* or baby or babies or newborn* or new-born*).tw,kf. Or exp *pregnant women/ or exp *Pregnancy/ or exp *Pregnancy Complications/ or exp *Obstetrics/ or exp *Breast Feeding/ OR<br>exp *Maternal Health Services/ or (breast-feeding education or parturition or ante natal or antenatal* or pre natal* or prenatal* or puerper* or postnatal* or postpartum or post partum or post natal* or peripartum or peri partum or prepregnancy or pre pregnancy or preconception* or pre conception* or periconception* or peri conception* or birth or childbirth or breastfe* or breast fe* or lactation* or stillbirth* or miscarriage* or pregnancy or pregnancies or pregnant or perinatal or peri-natal or mother or mothers or matern*).tw,kf.	((semi-structured or semistructured or unstructured or informal or in-depth or indepth or face-to-face or structured or guide) adj3 (interview* or discussion* or questionnaire* or survey*)).ti,ab. or (focus-group* or qualitative* or ethnograph* or fieldwork or field-work or key-informant).tw,kf. or interviews as topic/ or focus groups/ or narration/ or exp qualitative research/ or exp "surveys and questionnaires"/ NOT (case reports or comment or editorial or guideline or letter or practice guideline)	(Australia or Brisbane or Canberra or Sydney or Melbourne or Adelaide or Hobart or Perth or Darwin or Queensland or ACT or Australian-Capital-Territory or New South Wales or NSW or Victoria or Tasmania or Northern-Territory).tw,kf,in. or exp Australia/
**EMBASE**	coronavirus disease 2019/ or SARS coronavirus/ or experimental coronavirus disease 2019/ or exp severe acute respiratory syndrome coronavirus 2/ OR<br>(2019-novel or 2019nCoV or 2019-nCoV or COVID-19 or COVID19 or COVID-2019 or COVID2019 or CONVID-19 or CONVID19 or CORVID-19 or CORVID19 or CoV2 or CoV-2 or HCoV* or Ncov* or Ncorona* or Ncorono* or NcovChina* or NcovChinese* or NcovHubei* or NcovWuhan* or SARS2 or SARS-2 or SARScoronavirus2 or SARScoronavirus-2 or SARScoronovirus2 or SARScoronovirus-2 or SARSCov19 or SARSCov-19 or SARS-CoV-2 or SARSCoV-2 or SARSCoV2 or WN-CoV or WNCoV or wuhan-virus).tw,kf,dq,ot. OR<br>((pneumonia* or outbreak* or respiratory-illness* or respiratory-disease* or respiratory-symptom* or seafood-market* or food-market* or wildlife) and (Wuhan or China or Chinese or Hubei or Huanan)).tw,kf,dq,ot. OR<br>((new or novel or nouveau or "19" or "2019" or Wuhan or Hubei or Huanan or China or Chinese) adj3 (coronavirus* or corona virus* or betacoronavirus* or CoV or HCoV)).ti,ab,kf,dq,ot.. OR<br>(longCOVID* or postCOVID* or postcoronavirus* or postSARS*).ti,ab,kf,ot. OR<br>(*coronavirinae/ or *betacoronavirus/ or *coronavirus infections/) and (*disease outbreaks/ or *epidemics/ or *pandemics/) OR<br>((coronavirus* or corona-virus* or betacoronavirus*) adj3 (pandemic* or epidemic* or outbreak* or crisis)). tw,kf,dq,ot. OR<br>Severe-acute-respiratory-syndrome-coronavirus-2.hw OR<br>Coronavirus-disease-2019.hw	fetus/ or exp infant/ or prematurity/ or (fetal or foetal or fetus or foetus or preterm or pre-term or prematur* or infan* or neonat* or baby or babies or newborn* or new-born*).tw,kf,dq.OR<br>exp PREGNANCY/ or exp PREGNANCY DISORDER/ or exp OBSTETRIC PROCEDURE/ or exp BREAST FEEDING/ or exp BREAST FEEDING EDUCATION/ or exp BIRTH/ or exp CHILDBIRTH/ or maternal health service/ or (breast-feeding education or parturition or ante natal or antenatal* or pre natal* or prenatal* or puerper* or postnatal* or postpartum or post partum or post natal* or peripartum or peri partum or prepregnancy or pre pregnancy or preconception* or pre conception* or periconception* or peri conception* or birth or childbirth or breastfe* or breast fe* or lactation* or stillbirth* or miscarriage* or pregnancy or pregnancies or pregnant or perinatal or peri-natal or mother or mothers or matern*).tw,kf,dq.	((semi-structured or semistructured or unstructured or informal or in-depth or indepth or face-to-face or structured or guide) adj3 (interview* or discussion* or questionnaire* or survey*)).ti,ab. or (focus-group* or qualitative* or ethnograph* or fieldwork or field-work or key-informant).tw,kf,dq. or exp interview/ or verbal communication/ or conversation/ or exp qualitative research/ or health care survey/ or exp questionnaire/	(Australia or Brisbane or Canberra or Sydney or Melbourne or Adelaide or Hobart or Perth or Darwin or Queensland or ACT or Australian-Capital-Territory or New South Wales or NSW or Victoria or Tasmania or Northern-Territory).tw,kf,dq,in. or exp Australia/

**PubMed**	((((“2019-novel” OR “2019nCoV” OR “2019-nCoV” OR “COVID-19” OR “COVID19” OR “COVID-2019” OR “COVID2019” OR “CONVID-19” OR “CONVID19” OR “CORVID-19” OR “CORVID19” OR “CoV2” OR “CoV-2” OR “HCoV*” OR “Ncov*” OR “Ncorona*” OR “Ncorono*” OR “NcovChina*” OR “NcovChinese*” OR “NcovHubei*” OR “NcovWuhan*” OR “SARS2” OR “SARS-2” OR “SARScoronavirus2” OR “SARScoronavirus-2” OR “SARScoronovirus2” OR “SARScoronovirus-2” OR “SARSCov19” OR “SARSCov-19” OR “SARS-CoV-2” OR “SARSCoV-2” OR “SARSCoV2” OR “WN-CoV” OR “WNCoV” OR “wuhan-virus”) OR ((“pneumonia*” OR “outbreak*” OR “respiratory-illness*” OR “respiratory-disease*” OR “respiratory-symptom*” OR “seafood-market*” OR “food-market*” OR “wildlife”) AND (“Wuhan” OR “China” OR “Chinese” OR “Hubei” OR “Huanan”)) OR ((“new” OR “novel” OR “nouveau” OR “19” OR “2019” OR “Wuhan” OR “Hubei” OR “Huanan” OR “China” OR “Chinese”) AND (“coronavirus*” OR “corona virus*” OR “betacoronavirus*” OR “CoV” OR “HCoV”)) OR (“longCOVID*” OR “postCOVID*” OR “postcoronavirus*” OR “postSARS*”) OR ((“coronavirus*” OR “corona-virus*” OR “betacoronavirus*”) AND (“pandemic*” OR “epidemic*” OR “outbreak*” OR “crisis”)))	((“fetal”[Title/Abstract] OR “foetal”[Title/Abstract] OR “fetus”[Title/Abstract] OR “foetus”[Title/Abstract] OR “preterm”[Title/Abstract] OR “pre-term”[Title/Abstract] OR “prematur*”[Title/Abstract] OR “infan*”[Title/Abstract] OR “neonat*”[Title/Abstract] OR “baby”[Title/Abstract] OR “babies”[Title/Abstract] OR “newborn*”[Title/Abstract] OR “new-born*”[Title/Abstract] OR) OR (“parturition”[Title/Abstract] OR “ante natal”[Title/Abstract] OR “antenatal*”[Title/Abstract] OR “pre natal*”[Title/Abstract] OR “prenatal*”[Title/Abstract] OR “puerper*”[Title/Abstract] OR “postnatal*”[Title/Abstract] OR “post-natal*”[Title/Abstract] OR “postpartum”[Title/Abstract] OR “post-partum”[Title/Abstract] OR “peripartum”[Title/Abstract] OR “peri partum”[Title/Abstract] OR “prepregnancy”[Title/Abstract] OR “pre pregnancy”[Title/Abstract] OR “preconception*”[Title/Abstract] OR “pre conception*”[Title/Abstract] OR “periconception*”[Title/Abstract] OR “peri conception*”[Title/Abstract] OR “birth”[Title/Abstract] OR “CHILDbirth”[Title/Abstract] OR “breastfe*”[Title/Abstract] OR “breast fe*”[Title/Abstract] OR “lactation*”[Title/Abstract] OR “stillbirth*”[Title/Abstract] OR “miscarriage*”[Title/Abstract] OR “pregnancy”[Title/Abstract] OR “pregnancies”[Title/Abstract] OR “pregnant”[Title/Abstract] OR “perinatal”[Title/Abstract] OR “peri-natal”[Title/Abstract] OR “mother”[Title/Abstract] OR “mothers”[Title/Abstract] OR “matern*”[Title/Abstract] OR “Obstetric*”[Title/Abstract] OR “birth”[Title/Abstract] OR “childbirth”[Title/Abstract]))	(“semi-structured” [Title/Abstract] OR “semistructured” [Title/Abstract] OR “unstructured” [Title/Abstract] OR “informal” [Title/Abstract] OR “in-depth” [Title/Abstract] OR “indepth” [Title/Abstract] OR “face-to-face” [Title/Abstract] OR “structured” [Title/Abstract] OR “guide”) [Title/Abstract] AND (“interview*” [Title/Abstract] OR “discussion*”) [Title/Abstract] OR “questionnaire*” [Title/Abstract] OR “survey*” [Title/Abstract] OR “focus group*” [Title/Abstract] OR “qualitative*”[Title/Abstract] OR “ethnograph*” [Title/Abstract] OR “fieldwork” [Title/Abstract] OR “field-work” [Title/Abstract] OR “key-informant” [Title/Abstract] OR “narration” [Title/Abstract] OR “qualitative-research” [Title/Abstract]	“Australia” [Title/Abstract] OR “Brisbane” [Title/Abstract] OR “Canberra” [Title/Abstract] OR “Sydney” [Title/Abstract] OR “Melbourne” [Title/Abstract] OR “Adelaide” [Title/Abstract] OR “Hobart” [Title/Abstract] OR “Perth” [Title/Abstract] OR “Darwin” [Title/Abstract] OR “Queensland” [Title/Abstract] OR “ACT” [Title/Abstract] OR “Australian-Capital-Territory” [Title/Abstract] OR “New South Wales” [Title/Abstract] OR “NSW” [Title/Abstract] OR “Victoria” [Title/Abstract] OR “Tasmania” [Title/Abstract] OR “Northern-Territory” [Title/Abstract] NOTNLM OR publisher[sb] OR inprocess[sb] OR pubmednotmedline[sb] OR indatareview[sb] OR pubstatusaheadofprint
**Web of Science**	(All fields) “2019-novel” OR “2019nCoV” OR “2019-nCoV” OR “COVID-19” OR “COVID19” OR “COVID-2019” OR “COVID2019” OR “CONVID-19” OR “CONVID19” OR “CORVID-19” OR “CORVID19” OR “CoV2” OR “CoV-2” OR “HCoV*” OR “Ncov*” OR “Ncorona*” OR “Ncorono*” OR “NcovChina*” OR “NcovChinese*” OR “NcovHubei*” OR “NcovWuhan*” OR “SARS2” OR “SARS-2” OR “SARScoronavirus2” OR “SARScoronavirus-2” OR “SARScoronovirus2” OR “SARScoronovirus-2” OR “SARSCov19” OR “SARSCov-19” OR	(Title/abstract) “fetal” OR “foetal” OR “fetus” OR “foetus” OR “preterm” OR “pre-term” OR “prematur*” OR “infan*” OR “neonat*” OR “baby” OR “babies” OR “newborn*” OR “new-born*” OR “parturition” OR “ante-natal” OR “antenatal*” OR “pre-natal*” OR “prenatal*” OR “puerper*” OR “postnatal*” OR “post-	(Title/abstract) (“semi-structured” OR “semistructured” OR “unstructured” OR “informal” OR “in-depth” OR “indepth” OR “face-to-face” OR	(Title/abstract) “Australia” OR “Brisbane” OR “Canberra” OR “Sydney” OR “Melbourne” OR “Adelaide” OR “Hobart” OR “Perth” OR “Darwin” OR “Queensland” OR “ACT” OR

	"SARS-CoV-2" OR "SARSCoV-2" OR "SARSCoV2" OR "WN-CoV" OR "WNCoV" OR "wuhan-virus" OR "2019-novel" OR "2019nCoV" OR "2019-nCoV" OR "COVID-19" OR "COVID19" OR "COVID-2019" OR "COVID2019" OR "CONVID-19" OR "CONVID19" OR "CORVID-19" OR "CORVID19" OR "CoV2" OR "CoV-2" OR "HCoV*" OR "Ncov*" OR "Ncorona*" OR "Ncorono*" OR "NcovChina*" OR "NcovChinese*" OR "NcovHubei*" OR "NcovWuhan*" OR "SARS2" OR "SARS-2" OR "SARScoronavirus2" OR "SARScoronavirus-2" OR "SARScoronovirus2" OR "SARScoronovirus-2" OR "SARSCov19" OR "SARSCov-19" OR "SARS-CoV-2" OR "SARSCoV-2" OR "SARSCoV2" OR "WN-CoV" OR "WNCoV" OR "wuhan-virus" OR ("pneumonia*" OR "outbreak*" OR "respiratory-illness*" OR "respiratory-disease*" OR "respiratory-symptom*" OR "seafood-market*" OR "food-market*" OR "wildlife") AND ("Wuhan" OR "China" OR "Chinese" OR "Hubei" OR "Huanan") OR ("pneumonia*" OR "outbreak*" OR "respiratory-illness*" OR "respiratory-disease*" OR "respiratory-symptom*" OR "seafood-market*" OR "food-market*" OR "wildlife") AND ("Wuhan" OR "China" OR "Chinese" OR "Hubei" OR "Huanan") OR("new" OR "novel" OR "nouveau" OR "19" OR "2019" OR "Wuhan" OR "Hubei" OR "Huanan" OR "China" OR "Chinese") AND ("coronavirus*" OR "corona virus*" OR "betacoronavirus*" OR "CoV" OR "HCoV") OR("new" OR "novel" OR "nouveau" OR "19" OR "2019" OR "Wuhan" OR "Hubei" OR "Huanan" OR "China" OR "Chinese") AND ("coronavirus*" OR "corona virus*" OR "betacoronavirus*" OR "CoV" OR "HCoV") OR "longCOVID*" OR "postCOVID*" OR "postcoronavirus*" OR "postSARS*" OR "longCOVID*" OR "postCOVID*" OR "postcoronavirus*" OR "postSARS*" OR ("coronavirus*" OR "corona-virus*" OR "betacoronavirus*") AND ("pandemic*" OR "epidemic*" OR "outbreak*" OR "crisis") OR ("coronavirus*" OR "corona-virus*" OR "betacoronavirus*") AND ("pandemic*" OR "epidemic*" OR "outbreak*" OR "crisis")	natal*" OR "postpartum" OR "post-partum" OR "peripartum" OR "peri-partum" OR "prepregnancy" OR "preconception*" OR "pre-conception*" OR "periconception*" OR "peri-conception*" OR "breastfe*" OR "breast-fe*" OR "lactation*" OR "stillbirth*" OR "miscarriage*" OR "pregnancy" OR "pregnancies" OR "pregnant" OR "perinatal" OR "peri-natal" OR "mother" OR "mothers" OR "matern*" OR "obstetric*" OR "birth" OR "childbirth"	"structured" OR "guide") AND ("interview*" OR "discussion*") OR "questionnaire*" OR "survey*" OR "focus group*" OR "qualitative*" OR "ethnograph*" OR "fieldwork" OR "field-work" OR "key-informant" OR "narration" OR "qualitative-research"	"Australian-Capital-Territory" OR "New South Wales" OR "NSW" OR "Victoria" OR "Tasmania" OR "Northern-Territory

Appendix 4 Table of Evidence (extended)

Theme	Examples
1)*"No one can give you any answers":* Provision of information was inadequate in supporting women to make health-related decisions	• Atchan 2023 "Yeah, and I guess for me I think that the biggest thing is as a new mum you kind of look to people, you look to things, or you Google just trying to work things out and you get the advice, but because it's all telehealth, it's all disconnected, there's that how is missing, like if that makes sense. I did spend a lot of time, like I wasted a lot of time Googling and reading forums and blogs. Some stuff was really good, but also some stuff that was definitely not useful on everything from breastfeeding to trying to make my baby sleep to sleep training to co-sleeping to mixed feeding, tongue ties…everything that is out there in Google, I read it."<br>• Atmuri 2022 "I wish that I knew, had a picture in my head, of what I was going to be walking into...I guess there's a little bit of anxiety about getting lost and just, yeah, the idea of not knowing is a little disappointing".<br>• Atmuri 2022 "The birthing classes were cancelled...so I have looked up the information online and I don't know if that's everything that was there...it's hard to know when you're doing the research yourself as opposed to in a class."<br>• Atmuri 2022 "I'd like to have a water birth and I don't know if that's still possible. I don't know if water births are allowed again? Or being in the water during labour?"<br>• Atmuri 2022 "You do feel a little bit stressed in there, and I think probably one thing that maybe could be improved is just that extra information of what you are doing with the COVID stuff in terms of precautions, what it's going to look like when I come in to have bubs, just what to expect."<br>• Atmuri 2022 "What happens when you come in? Because obviously there's not been hospital tours … I wouldn't even know where to go. A video online on the website or something that you can go on and get a tour may be helpful, starting from outside so you know where you're going."<br>• Caddy 2023 "No that [hospital restrictions] was communicated terribly. . . or it just wasn't communicated at all. before I went in to have the baby I asked about the restrictions, and the nurse on maternity just didn't even know...because I didn't have a husband coming in you know, so what does [my] mum count as and can she bring back my son and they just were clueless."<br>• Caddy 2023 "It made it a little bit more stressful than it needed to be. Not knowing, sort of feeling like you're in the dark a little bit and there's no one who's in your corner."<br>• Caddy 2023 "it had an impact to the birthing experience, because it [lack of information] made probably an experience [birth] more negative…because I had no idea what to expect, with COVID regulations."<br>• Caddy 2023 "There hasn't actually been any proactive messaging to me about COVID-19, especially regarding the vaccines there's been nothing mentioned to me or discussed with me or with any of the health providers, if that would affect me, or even if I would be able to get the vaccine."<br>• Caddy 2023 "I just feel like, tell us what's going on, let us know that you're dealing with it, or let us know what is that it's being investigated or whatever, but don't pretend it's not happening."<br>• Caddy 2023 "So there was no access to birthing classes, any of that sort of stuff. . .we were just winging it basically."<br>• Caddy 2023 "I think all my information about COVID, and pregnancy probably came through her [my obstetrician]...she talked a lot about how I could manage being pregnant in this situation and keeping safe."<br>• Caddy 2023 "I would pass New South Wales Health [department]...I don't think they've been very good actually at presenting like digestible information".<br>• Caddy 2023 "What I did seek from missing out on antenatal classes lactation consultants and midwives that are now providing their services through Instagram, and they will post, ask me anything type of stories."<br>• Caddy 2023 "People saying that it [vaccine] increased the risk of miscarriage and stuff. I mean, just the sorts of things you'd kind of expect to be said in terms of misinformation."<br>• Caddy 2023 "I follow the hospital on Instagram. And they're pretty good, it's sort of communicating changes very, in a very timely manner. So, they will, anytime the rules changed, you know, within a couple of hours, they would have posted on Instagram saying, here's the new rule. And I think that was quite good."<br>• Caddy 2023 "I googled the s—t out of, you know, what would happen if I got COVID-19 while I was pregnant. What would happen if I ended up having it [COVID-19] while I was going in to give birth...would they take her away if I was diagnosed positive."<br>• Caddy 2023 "the information in the public domain wasn't really that much...I guess that's what Facebook kind of gave me is that broader social network to ask questions or to at least have information that wasn't in the general publications."<br>• Caddy 2023 "sometimes because you have to call the antenatal place and you're usually on hold for like an hour, so we just check there [Facebook group] first because people answer faster."<br>• Caddy 2023 "Probably not sourcing it out, it would just pop up on Facebook, because I've obviously googled something...So then I would just click on something from there"

	• Davis 2021 “It’s not until you’re in that deep, dark place that you need that help and someone can tell you, whereas if I’d known about it before, I might not have gotten to that point.”<br>• Davis 2021 “[The information]…it’s just too complicated for a normal person to understand it. We’re not medics, we’re not in that field, so you’re just like, “What exactly are you trying to say?”<br>• Davis 2021 “When they cancelled everything, it took weeks for them to do anything online and then it was pages of reading, there’s no videos, no nothing, and then you have all these questions, and you try to call to find out some answers and no one can give you any answers and there was no one to talk to.”<br>• Davis 2021 “It would have been useful to maybe have some generic information that went out to women in that situation, if you were pregnant, [for example], “It’s early days. We don’t know what the impact could be”…Statements from a medical professional to put people’s minds at ease.”<br>• Davis 2021 “I just feel like pregnant women weren’t supported through the pandemic.”<br>• Davis 2021 “You shouldn’t have to be going private to get this information because it’s really just standard information that anyone should be getting...you shouldn’t have to pay that amount of money to get what’s really a basic human right to understand and to get.”<br>• Oliver 2022 ‘We [pregnant women] were being told ‘that’ and now we’re being told ‘this’. What if we get told something in a few months’ time that contradicts the action we’ve taken [getting vaccinated]? ...I’m going to wait and find out what it is next.”<br>• Oliver 2022 ‘‘It’s hard to go by the media. They blow everything out of proportion, or they cover up a lot of things. ...the less you know the better when it comes to the news.”<br>• Sweet 2021 “Because there wasn’t a lot of information around whether COVID was more dangerous for people that were pregnant or not. There was contradicting statements saying yes it can get to your baby through the womb and no it can’t, and so there was not much information that was 100% certain”.<br>• Sweet 2021 “I feel like I’ve needed to ask for more information, around… the early signs of labour and all that sort of stuff, just because I am a first-time mum, and I don’t know what to expect”<br>• Sweet 2021“I think it was just that anxiety…[so I was] trying to find out as much information as I could…I think there was a lot of anxiety on my part…I’d ask questions like ‘is this going to affect my pregnancy?’ and they’d say, ‘we just don’t know’.”<br>• Sweet 2021 “And when you’re not being supported it does amp [amplify] up that anxiety as well, about what’s happening in the world, and how’s it going to be when I go to hospital, can my husband come with me? What am I going to do with the other children? they’re not allowed to go to their grandparents at the moment … it just changes all the time … we’ll just roll with it and wing it”<br>• Sweet 2021 “I tend to sort of self-research a lot and I’d read the WHO [World Health Organisation] guidelines”.<br>• Sweet 2021 “the college of obstetrics and gynaecology, I’ve kept sort of up to date with their guidelines”.<br>• Sweet 2021 “I looked at the academic analysis that had been done on these six cases in Wuhan and I looked at things like [RANZCOG]”<br>• Sweet 2021 “my obstetrician was really good and said if you want more information, we’ve been accessing RANZCOG, and she said have a look there, they’ve got all the up-to-date stuff for pregnant people on their”.<br>• Sweet 2021 “There was a Facebook group that I was on that did a live interview with an obstetric professor answering questions about COVID which was really helpful”.<br>• Sweet 2021 “I think my obstetrician – she has like a social media page, she would publish [from] the college of obstetricians, she would post their latest guidance, because she has lots of anxious people”.<br>• Sweet 2021 “because you just can’t trust them – like you’ve got to decipher through what’s true and what’s not …Is that having a positive influence on me, and my mental and physical health, or not? And if it’s a no well, why am I engaging in this?”<br>• Sweet 2021 “just really on the news, which maybe isn’t the best source but just looking at what was coming up on the news”.<br>• Sweet 2021 “watching the live updates from the premier … I followed the government’s Facebook website updates daily”<br>• Sweet 2021 “the information was very rapidly changing, and I was obviously keeping up to date on Facebook and that seemed like everyone was sharing different updates”.<br>• Sweet 2021 “I felt like I always had to be on top of everything, and if I wasn’t going to be on top of it then things were going to get missed. So, I constantly felt like I had to advocate for me and the baby to make sure that things didn’t go awry…if it’d been my first, I could’ve got lost in the system quite easily”
2) *“Very isolated”* or *“It brought us closer”*: Social distancing restrictions caused major changes within women’s informal support networks	• Atchan 2023 “I struggled a lot, not badly struggled, but struggled a lot without that support and all the rest of it, and I was a fourth time mum, so I really had a lot of empathy for those first-time mums that you know, something–I just hope that if something like this happens again that there’s strategies in place that can be a bit more supportive in that way”<br>• Atchan 2023 “…it was a lot more peaceful, and I feel like I easily understand what [baby]’s needs were because we didn’t have the interruptions of visitors and it was a bit more predictable in what he needed, like when he needed a feed.”<br>• Atchan 2023 “I just think it’s been almost a blessing in disguise that everyone has given me space with my newborn baby and that you can relish that time with just your family, and I think it has encouraged me to feed her because I am at home.”

	• Atchan 2023 “I think it’s just made breastfeeding less worrying because you’re always at home…like you’re not out so you don’t have to worry about the logistics of it. Probably the biggest thing is probably the ease…so you’re not stressed about having to express and pack and cover yourself up.”<br>• Atchan 2023 ”And I now have enrolled to do a breastfeeding peer support counselling course. So, I am really excited about that, and I feel that is my thing to do.”<br>• Atchan 2023 “I struggled a lot, not badly struggled, but struggled a lot without that support and all the rest of it, and I was a fourth time mum, so I really had a lot of empathy for those first-time mums that you know, something – I just hope that if something like this happens again that there’s strategies in place that can be a bit more supportive in that way.”<br>• Atchan 2023 “This one woman had her baby in the lockdown and she said, “Are you a first time mum?”, and I was like “yeah” she’s like “Oh God…I couldn’t have done that! I couldn’t have had a baby for the first time in a lockdown.” She’s like “That’s amazing!” and I was like “Oh yeah, you know”. I just don’t know anything else; you know, this was my experience.<br>• Atmuri 2022 ”You can’t attend some yoga or birth classes so you can’t meet some other mums as well. You can’t ask advice or experience from them. Because of this, I have to do it by myself.”<br>• Atmuri 2022 “I hope I won’t be going through with depression because of less people around. Sometimes you just need family support or even a friend’s support, just to help you out a little bit. Because your first time ... you don’t really have any idea about anything.”<br>• Atmuri 2022 “I don’t think many people have even seen me showing ... you miss all that part of pregnancy a bit because we’re being locked down. Likewise, with work, we do video conferences, and you can’t really tell I’m pregnant. I guess you kind of miss a bit of the hubbub about being pregnant, the excitement.”<br>• Atmuri 2022 “Am I going to have a baby shower? Who knows? The people that I’m catching up with now, it’s the first time I’ve seen them since they found out I was pregnant and now I have a bump.”<br>• Atmuri 2022 “... As well moving forward I’m really keen for my mental health to be part of a mother’s group and I’m concerned that those mother’s groups might not be happening.”<br>• Caddy 2023 “There was actually three of us that were pregnant all at the same time ... if they found anything helpful, they’d send me a screenshot, or we often talked about like, it’s going to be okay kind of thing. ... It was hard for any family to be around us. It was hard for them to understand, they weren’t pregnant in a pandemic.”<br>• Davis 2021 “Just to be able to obviously talk to other people that were going through basically the same things just made it all a little bit easier.”<br>• Davis 2021 “Having those people that we’re going through the exact same as what I was was really helpful.”<br>• Davis 2021 “ being able to speak to other women in the same situation and they have their coping mechanism and what they’re doing, that might help as well.”<br>• Davis 2021 “It was quite sad that I couldn’t even share my pregnancy experience - as scary as it was, I couldn’t share that with anyone, and I feel like I missed out.”<br>• Davis 2021 “There’s a whole group of mothers out there that are just there by themselves.”<br>• Davis 2021“For six months, there’s a whole group of mothers from late February to March/April that were just forgotten about and those mothers, some of them now at nine months make no connections with any other mothers because they were just left.”<br>• Davis 2021 “I know a lot of us saw the benefits of a reduction in visitors...We were able to use coronavirus as an excuse just to stay in our little bubble.”<br>• Davis 2021 “In fact, if anything I didn’t feel any pressure to go out and see people, I could just relax at home ... I suppose the one good thing about not having everyone come over and visit was having that time to adjust to having a baby without lots of visitors and lots of expectations to host for people.”<br>• Davis 2021 “It’s quite nice being less sociable ... It’s quite nice not having all these different appointments booked in to see people. I think it just made everyone slow down a little bit.”<br>• Davis 2021 “It’s been really great that my husband had been able to work from home a lot throughout the year...So, if that’s one thing that we could keep, would be him to continue to work from home.”<br>• Hood 2021 “I felt very alone and very isolated...It triggers all those feelings of isolation and just endless hours at home by myself or with [child].”<br>• Hood 2021 “It was so hard because I see my parents quite a lot, and they refused to see me. My husband was at work and it was pretty much just me and the kids all day, every day.”<br>• Hood 2021 “So in particular with Corona, because we had been doing baby sensory classes we’re doing some online videos through the restrictions. So I did a little bit of that with him, which helped with the bonding at the time.”<br>• Hood 2021 “Even our physio exercise classes were on Zoom. We had our church mass on Zoom...They [devices] probably helped both of our mental states ... For me to be able to do some exercise and everything and just see that there was other people out there, that life was going on.”<br>• Hood 2021 “It helped alleviate a bit of our anxiety, just living through a pandemic...By having something to still be connected to the rest of the world I think it was stress relieving.”<br>• Hood 2021 “Martial arts went online. So we actually did that from home for a while there, which was pretty cool.”

	• Hood 2021“They [9 year old daughters and their friends] do bingo online with each other and that (you know, even just talking online), that was a whole new thing. And that’s a positive thing.”<br>• Hood 2021 “We basically didn’t leave the house for two months”<br>• Hood 2021 “We didn’t go to the library or rhyme time or catch up as much with other mums.”<br>• Hood 2021 “Because of the isolation restrictions it meant that we weren’t able to go and do the activities that we were doing such as baby sensory and Gymbaroo ... which meant being stuck at home which became very much a bit of a ground hog day.”<br>• Hood 2021 “I felt a bit sorry for him because it was going to be the time that he started doing his little groups and things at the beginning of COVID. And then they were all cancelled so he didn’t”<br>• Hood 2021 “My family’s all on the East coast [of Australia] so they haven’t been able to come over and visit for our birthday. And my mum is, this is her first grandchild, so she’s really missing seeing everything. She would have been over here at least once, maybe twice if it wasn’t Corona.”<br>• Hood 2021 “The mother’s group had only met a couple of times. And so to then just not meet, like our mother’s group essentially is kind of diminished because there wasn’t long enough, strong enough connections to keep it going. And I feel sorry for the people who didn’t get to join the mother’s group at all, because I desperately needed that support at the beginning.”<br>• Hood 2021 “We’ve become more attached because we’ve had to stay at home together.”<br>• Hood 2021 “It did affect our relationship. In a good way. We were quite close.”<br>• Hood 2021 “It actually made me more connected to him because I worked from home so I had time and he could see me throughout the day.”<br>• Hood 2021 “Since this COVID-19 started my husband is at home more, so that’s when I see that he’s getting more closer to his dad.”<br>• Hood 2021 “She [1 year old] was with him [father] twenty-four seven. So they did become more attached, which was quite nice.”<br>• Hood 2021 “I’d probably say it brought us all [mother, father and two children] closer to be honest because we had to entertain them as opposed to going out and entertaining them, like at the aquarium or the zoo. Like I had to entertain them at home. So yeah, I guess it brings that bond closer.”<br>• Hood 2021 “With my husband, he had to work from home for about six weeks. And because of that, it allowed us to go for a walk together in the morning and we were able to have lunch together. And that was actually really really nice. And that was definitely nice for the [marital] relationship.”<br>• Hood 2021 “It actually was a blessing in lots of ways as well. It was good family time and actually being able to properly interact with each other at home ... There was more free time to actually do things and activities. So probably in that way, it was probably better for our [family] relationship.”<br>• Hood 2021 “I think the Corona, talking to other people and feeling it for myself, it actually deepens the family bond because you realise that, either spending more time at home or just listening to other stories, it feels like: ‘This is important. I don’t need to go anywhere really.”<br>• Hood 2021 “It was nearly a welcome change because it gave us an excuse to stay at home ... You kind of had more time to yourself that you could concentrate on their development rather than rushing around trying to do these classes. And now I kind of realise after the fact that we probably do a little bit too much. It was probably nice to actually get to take the break, and less is more with babies. I think I’ve just learned that.”<br>• Hood 2021 “Before I used to go shopping because I didn’t know what to do with myself and I needed to get out of the house, and now I don’t have that need anymore and I feel like this is good, we can just be us...I think it brought us closer. The value of spending time together and that’s the time we’ve got, we should be spending together and enjoying it...It actually teaches you things, teaches you to embrace your family.”<br>• Hood 2021 “Coronavirus kind of reaffirmed the need for healthy habits and finding a nice balance. You know, finding, trying to find a balance between the benefits of using screen time, using screens to promote how you live rather than letting screen times dictate how you live.”<br>• Hood 2021 “We did a lot more ... We were throwing balls, riding bikes, playing board games, my little one would help me with sewing.”<br>• Hood 2021 “I bought some art supplies and things like that so we could do more activities together.”<br>• Hood 2021 “We would go for walks every day, and yeah, generally we try and do different activities.”<br>• Hood 2021“She just wasn’t so dependent on me anymore ... It’s been nice for him [husband] and most of the time I think it’s nice, but there’s still part of me that goes, well, she’s my little baby. I liked being her favourite. But you’ve got to do what you’ve got to do.”<br>• Hood 2021 “While we were all at home, we kind of made more of an effort to spend more time with the family. So we did a lot of board games and colouring in and activities and stuff rather than, we didn’t have much time on the tablets or TV or anything at all.”<br>• Hood 2021 “We had a number of family birthdays over Zoom”<br>• Hood 2021 “Using devices actually helped us to get in touch. We probably got in touch more often than we normally do, just to check on them [family in Italy] and [ask] “is everything all right and how are you coping being under lockdown?” I think with the rest of the family,

	actually devices and all the Skype messages and the emails helped, and sending photos of grandchildren to keep the spirits up and things like that. That was actually a good thing. I think it came handy and we used it in a positive way."<br>• Hood 2021 "We do more video calls now because we can't go and visit family in the Eastern States (of Australia)."<br>• Hood 2021 It was very bad in Italy and it was a bit overwhelming for our family back there. So we tried to call them a little bit more because they had the lockdown. So they had to stay home and we tried to be more close to them and call them more often. So maybe we called even more than once a day."<br>• Sweet 2021 "I sort of feel like I am going it alone."<br>• Sweet 2021 "It's been very impersonal, being pregnant for the first time, I find myself not having anyone to talk to and it does feel very alone. I've got my husband but he's not going through what I'm going through."<br>• Sweet 2021 "It was really rough for the first few weeks – my parents live a few hours away so they didn't meet him until he was 6 weeks old, and the plan had originally been that mum would come up and support us, obviously that didn't happen."<br>• Sweet 2021 "no one saw him in that whole 6 weeks, which is a little bit stressful for a first time mum."<br>• Sweet 2021 "the restrictions meant that we've lost some supports that we would've had otherwise."<br>• Sweet 2021 "I was planning to have some support from my family … when the baby was born but my parents couldn't make it from Canada … and my partner's parents were planning to come down too and they're only in Queensland."<br>• Sweet 2021 "… my social life with friends, it was so important for me, their help and support, because like we don't have family here and I have only friends, but I can't see them."<br>• Sweet 2021 "We brought my mum up here…and then they closed the regional borders,…so we had to send her home after three days, so I didn't have that support that we had planned".<br>• Sweet 2021 "We had to change our plans massively because my Mum was going to come down and look after her, which is not going to happen"<br>• Sweet 2021 "It was good to have COVID as an excuse not to have people over … because of the restrictions it's meant that I haven't had to politely decline people coming over, and everyone seeing the baby and wanting to cuddle her and all of that, I didn't have to deal with any of that "<br>• Sweet 2021 "At the same time it was also really nice to have a reason not to leave the house and to be able to say we're just going to stay home and have this quiet time as a family "<br>• Sweet 2021 "We just stayed in our little bubble and we all slept when he [baby] slept, and we watched movies and cuddled…and–we probably wouldn't have done all that…the pandemic made that part a lot nicer "<br>• Sweet 2021 "Well I guess because normally you'd go out and have coffee and just meet with other mums…there's been a lot of just staying at home…so it is quite lonely sometimes, but luckily there's Zoom and Skype and Facetime"<br>• Sweet 2021 "I think if I had been a first-time mum it would have been a lot harder – this time I knew what I needed and where to get that from,…I think definitely not knowing what services were around – how to access them and how they were being delivered differently that would have been a huge barrier".<br>• Sweet 2021 "I joined that group [on Facebook] and then found that me and other mums that had other babies were having to advise these first time mums, … 'hey that's not cool', like 'you need to call up the clinic and go get assessed if that's happening '… don't let them brush you off"<br>• Sweet 2021 "so there were a few groups that popped up [on Facebook] with women just trying to support each other during that time "<br>• Sweet 2021 "I rang my Mum, I can't see her either at the moment … I had this big meltdown; this is not how it was supposed to be. But I kind of just try to get on with it"<br>• Sweet 2021"there was even some [other women] volunteering to look after people's children when they were meant to have ultrasounds, people they'd never met, and they were like 'I've got no one to watch my kids and I can't take them to the ultrasound because of COVID', and people were like 'well where do live, I can watch your kids while you go'. It's like okay you're going to leave your kids with someone you've just met on a Facebook group because you're that desperate"<br>• Sweet 2021 "just this morning I caught up with my local mothers' group – so a lot of mums are using Facebook to arrange postcode mums' groups"

3) “*Have they seen enough of me?”:* Women felt unsupported during disruptions in maternal health services	• Atchan 2023 “I struggled a lot, not badly struggled, but struggled a lot without that support and all the rest of it, and I was a fourth time mum, so I really had a lot of empathy for those first-time mums that you know, something – I just hope that if something like this happens again that there’s strategies in place that can be a bit more supportive in that way.”<br>• Atchan 2023 “So you know like, first time Mum right, so I knew that if you had mastitis you were meant to get onto it really quickly, because I did not want to take antibiotics, and so like after waiting 2 h to have a teleconference about what I should do, just being told actually, can you just come in, I can’t see if you have mastitis or not.”<br>• Atchan 2023 “Yeah so we had midwives come to my house for 2 or 3 visits over the weekend, because I was having trouble with breastfeeding. And then I noticed that he was jaundiced, and I finally got the lactation lady when he was 8 days old, and she said that he was jaundiced, and we needed to go back into hospital for the night … We had a few clinic visits after that, but then they stopped because of COVID, so it was all on the phone. I couldn’t get him weighed which was really hard because he was failing to thrive as well.<br>• Atchan 2023 “But it would have been awesome to have seen a Lactation Consultant to check out our latch early on because I desperately started Googling, has he got a tongue-tie, has he got a lip-tie, do I need to see an Osteopath? But I can’t go there either. Or a Chiropractor? But I can’t go anywhere and my mum can’t come around, no one.”<br>• Atchan 2023 “I struggled with feeding her and she lost weight and then there was just no one to help you – like the consultants were only via Zoom which was quite – not very helpful.”<br>• Atchan 2023 “…lactation needs to be face-to-face – like the Zoom was quite tricky and I’ve had previous Zoom meetings when even like I can’t really tell but I think she’s ok – do you know what I mean and by the time we got to face-to-face it was almost too late.”<br>• Atchan 2023 “And then everything sort of changed where they couldn’t then come and visit me anymore, it was all over the telephone, so I couldn’t go and get him weighed, I didn’t know how he was going weight wise with what we were doing, so I couldn’t get anyone to come and check to see how we were breastfeeding, and yeah with three other children at home it was quite full on really.”<br>• Atchan 2023 “I was just like – anyway I thought I can afford it, I’m going to call a private lactation consultant because I’m just not getting this support. It was the best decision…of my life”<br>• Atchan 2023 “The nurse who was on explained that she felt more comfortable for me and her to wear full PPE. I was given the baby and given him to feed. I was also given a syringe to express colostrum which I couldn’t do and that was very awkward because they were trying to stay apart and tell me what to do. So, in the end they did end up coming to help, but it was very – I wasn’t really taught how to do it; it was just a ‘let’s get this done and get out of here’ kind of feeling.“<br>• Atchan 2023 “I had every intention of [exclusive] breastfeeding, I feel like if Covid hadn’t happened we would’ve got this [breastfeeding] issue sorted properly, and I probably wouldn’t be mix feeding. But I don’t know what they [health providers] would’ve been able to do, but I feel like we didn’t get that chance to even explore it.”<br>• Atchan 2023 “When I came home from hospital, I wanted a breast pump from my midwife, and I wanted my brother to go and pick up the breast pump from her, but she wasn’t going to meet him because she didn’t know him … it kind of had to be contactless, which was a little bit annoying and frustrating trying to get something like a breast pump when I needed it”<br>• Atchan 2023 “… in the meantime, I’d been to the maternal child health nurse and she’s a lactation consultant too but she was under really strict guidelines by the Dept of Health that she only had 15 min so she basically said you have to top her up with a bottle you have no choice – because she couldn’t help and of course once you start doing that it’s just you know downhill basically.”<br>• Atchan 2023 “The Maternal and Child Health nurse, they didn’t do a two-week visit, so then you had to go into the clinic and by the time you get to the clinic she’d lost weight and her feeding was a disaster and you know because the appointment was only 15 min they can’t help you with your feeding, … so I found all that quite unsupportive during that time.”<br>• Atchan 2023 “…they’ve all got postnatal depression because they haven’t had any support of breastfeeding or been able to breastfeed as a result of that. And so, they’ve actually given up on breastfeeding, it’s made them feel really heavy and it’s made me really sad as well because you know normally you hear something like that and you just head up to their house and have a chat…but now you can’t, you can’t go anywhere with anyone.”<br>• Atchan 2023 “I was so upset. And like anxious you know like knowing, knowing how important the early weeks are. Knowing that I couldn’t get help. Knowing how badly I wanted to breastfeed. It’s like, it’s like my life depended on being able to breastfeed him. And also, like and anxious that my anxiety was affecting my supply. Anxious and it was just this like spiral and [partner’s name] couldn’t really get it, like he thought ’you’re kind of killing yourself to do this, there’s a really easy solution like if he just needs to be fed we just give him a bottle no problem’<br>• Atchan 2023 “I look back and it’s just this blur of obsession with trying to get breastfeeding going and just it was like my life depended on it. And just so, so anxious you know like. Just the feeling that you can’t get help you know and just the fact that first time mums, or any mums in the postnatal period - that physical contact wasn’t - like it was decided that wasn’t really important. You know like you can have someone there for your birth but.they just send you home.to figure out a really hard thing.”<br>• Atchan 2023 “That blue book is like pretty much empty because you know we’ve been

	weighing her by weighing me and then weighing her and you know, like her clothes are getting tighter so we're like she's doing well. So we're like okay."<br>• Atmuri 2022 "If I have a birth that requires a bit more intervention ... that requires a hospital stay, then I do think that would affect us ... that could have quite a big impact on visitors and support."<br>• Atmuri 2022 "I was a little bit worried about being sent home early...because this is my first baby. I'm also going home on my own...I don't have a partner to help me or to help me look after me or the baby or anything...and even just breastfeeding - so that was a bit of a worry."<br>• Atmuri 2022 "What if something happens with me not straight away but it could be a lot of medical challenges and obviously I don't want newborn to be exposed to situation where it may get affected."<br>• Atmuri 2022 "I just want to know, if anything happened to me that I could come to the hospital straightaway and they can treat me straight away."<br>• Atmuri 2022 "Sometimes I've walked out and thought, 'Oh, I meant to ask that', but I didn't really get time because I felt a bit of pressure to hurry up."<br>• Atmuri 2022 "You don't have that physical connection with someone or just being, knowing that they can physically see you and assess you. I had a miscarriage only a few months before I actually fell pregnant again ... I just feel like that being delayed (the physical) it just made me more anxious."<br>• Atmuri 2022 "I would say, if it was closer towards my due date, it would be very concerning for me ... am I getting the amount of scans that I need to or the amount of check-ups that I need to in person? Have they seen enough of me or got enough information to be able to make a good call around the stage?"<br>• Atmuri 2022 "What happens when you come in? Because obviously there's not been hospital tours...I wouldn't even know where to go. A video online on the website or something that you can go on and get a tour may be helpful, starting from outside so you know where you're going."<br>• Atmuri 2022 "Attending maternal child health appointments – I don't know if they're over the phone or face to face ... As well moving forward I'm really keen for my mental health to be part of a mother's group and I'm concerned that those mother's groups might not be happening."<br>• Atmuri 2022 "I wish that I knew, had a picture in my head, of what I was going to be walking into…I guess there's a little bit of anxiety about getting lost, yeah, the idea of not knowing is a little disappointing."<br>• Caddy 2023 "I didn't have the normal maternal and child health visits, how do you care for a newborn...what do you do when your child has a fever and you're in lockdown....I just wanted her [child] to see a GP but she couldn't until you've had a COVID test…and back then they were taking four to five days, what's happened to your freaking baby in the meantime?"<br>• Caddy 2023 "So there was no access to birthing classes, any of that sort of stuff. ... we were just winging it basically."<br>• Davis 2021 "I was really anxious. I did a calm birth course to try and help me just get some that sense of control back in a world where everything was so uncertain. So, I was just really stressed out ... I just feel like pregnant women weren't supported through the pandemic …You're just thrown in the complete deep end because you don't have regular support that you'd normally have."<br>• Davis 2021 "It's not until you're in that deep, dark place that you need that help and someone can tell you, whereas if I'd known about it before, I might not have gotten to that point."<br>• Davis 2021 "There was no face-to-face and just calling someone on the phone, it felt it was a bit impersonal. So, I relied heavily on my family to deal with my, you know, my anxiety and my outbursts and just being frightened for those few weeks."<br>• Davis 2021 "That (delay of 2-3 weeks to see a Child Health Nurse) doesn't help me when I've got something I'm worried about right now"<br>• Davis 2021: "... it's a lot more stressful not knowing has the baby got enough weight or are they going okay or even just having that check-in."<br>• Davis 2021 "I think when you're pregnant, it's very much that you want to show them physically what you're concerned about."<br>• Davis 2021 "I didn't have one successful phone call appointment."<br>• Davis 2021 "I think that initial phone conversation when my anxiety levels are quite high, I just shut it down and then didn't access services for a while."<br>• Davis 2021 "When they cancelled everything, it took weeks for them to do anything online and then it was pages of reading, there's no videos, no nothing, and then you have all these questions, and you try to call to find out some answers and no one can give you any answers and there was no one to talk to."<br>• Davis 2021 "To do it alone is terrifying"<br>• Sweet 2021 "I had to push quite hard to get what was going to work for us and just even to have it seems common sense to me, to have support in place, but apparently that's optional or not a necessity to them"?<br>• Sweet 2021 "my partner's not able to come with me which is a bit crap [bad]"<br>• Sweet 2021 ""it was daunting, I had to go to the hospital quite a lot at the last stage for monitoring and he [partner] wasn't allowed to be there for any of that, so that was pretty stressful, I just felt quite alone…I'm sitting in this hospital waiting to make sure that my baby's okay, and I have nobody to support me"<br>• Sweet 2021 "there was a lot of concern around what I would do with my children when I

	went into labour because the plan we had was for them to go with my parents but my parents are in their seventies"<br>• Sweet 2021 "we knew that my daughter wouldn't be able to visit in hospital which is what we had envisioned … but instead we kind of reframed all of that to be like it will just be us and we'll sort of see how we go from there"<br>• Sweet 2021 "because with all these restrictions…my husband can't come for labour…because someone has to look after our son"<br>• Sweet 2021 "luckily for me I am a health worker … and was able to get my husband in for that first scan just for 5 minutes at the end. But that was pretty stressful, and a bit of an ordeal, and probably took away from the moment of being first-time parents"<br>• Sweet 2021 "I couldn't have my sister there who was meant to be my second support person, … it was more just that I'd be alone, that was my biggest concern"<br>• Sweet 2021 "the scans my partner couldn't come with me … again I just felt alone like I couldn't share that with anyone … just to come to an appointment with me would have been good support, … it just does feel lonely"<br>• Sweet 2021 "I really needed his support and I'm glad that he was able to come in. If he wasn't able at that time, I think it would've been a much more traumatic experience not having him there"<br>• Sweet 2021"it was all negative kind of how you had to deal with everything and the stresses of all of that. I think seeing a maternal nurse in person and having a chat is very important."
4) "*All you want to do is keep safe"*: Safeguarding family from SARS-CoV-2 added cognitive strain to women's daily decision-making and routine.	• Atchan 2023 "I'm always a big advocate for breastfeeding and had planned to breastfeed him. I guess maybe even more so reinforced that I wanted to breastfeed him because of the transferred immunity in some sense if there was any."<br>• Atmuri 2022 "I think at the start there was so little understanding of how it could affect pregnant women. And I was hearing awful things where it wouldn't affect the child and then it could affect the child. A lack of understanding around risks was probably a little bit anxious for me."<br>• Atmuri 2022 "You do worry. You are concerned that everyone's doing the right thing, can I go to the shops, am I putting the baby at risk?"<br>• Atmuri 2022 "I'm giving birth in a hospital where sick people are. And it's probably – if people have COVID – they would be in the hospital. Just being in that same environment with a newborn is definitely a bit daunting."<br>• Atmuri 2022 "I actually stopped going to work ... I'm a kindergarten teacher and it was just recommended that given the limited research on what would happen."<br>• Atmuri 2022 "I always have a slight fear when I go into the hospital…that my husband – he'll get a temperature at delivery and won't be able to join me"<br>• Caddy 2023 "There's a lot of fear. What happens if I do get COVID? Is that gonna affect my pregnancy?...how's that gonna affect the baby potentially?"<br>• Caddy 2023 "I googled the s—t out of, you know, what would happen if I got COVID-19 while I was pregnant. What would happen if I ended up having it [COVID-19] while I was going in to give birth...would they take her away if I was diagnosed positive."<br>• Hood 2021 "It did become very stressful because we were worried. Obviously through all the media with everything going on which was all coming through the devices that we were going to, he was going to lose his job and it was going to be the end of the world. So it became very stressful for all of us, finding articles and sending it to him, you know, and him, us wanting to protect our son, make sure that we will have food, you know?"<br>• Hood 2021 "When it was quite bad in Western Australia around March/April, we just had to take him out of daycare for that two months and we would just stay at home"<br>• Hood 2021 "We didn't send our son to daycare because my parents came over from abroad and they are 70 years old, so we didn't want to take a risk."<br>• Hood 2021 "We haven't re-joined any of our classes that we did before. So it's still a bit, we're still a bit wary when we go out."<br>• Hood 2021 "Even before they started asking people to stay at home, I decided that's one thing that we could do for the community is to stay home as much as possible. So we probably, we did stay home more and we are still staying home more."<br>• Hood 2021 "Before COVID happened we had a busier week where we had swimming lessons and we would go to a dance class. But once that all stopped we just haven't got back into it."<br>• Sweet 2021 "I think it's because it was unknown. … I was feeling anxious and scared".<br>• Sweet 2021 "all you want to do is keep safe"<br>• Sweet 2021 "we kind of came up with our own plan to stay safe"<br>• Sweet 2021 "I wasn't hugging, I wasn't touching, they weren't touching me, they weren't touching my belly, there was none of that"<br>• Sweet 2021"being better with our hygiene, especially with the kids, we've put sanitiser near the door, so everyone bathes in it on the way in … we wash them [children] after childcare before going into our house … just being really good with them about touching and being around the baby"<br>• Sweet 2021 "we'd actually pulled our kids out a week earlier from school, because we kind of saw the writing on the wall, we've got a baby coming, obviously we were like we don't want to risk it"<br>• Sweet 2021 "I knew before he was born that I didn't want him meeting people straight away – I wanted to continue with social distancing even with family, grandparents – I was quite panicked about it"

	• Sweet 2021 “my husband sort of put me in lockdown about 2 months before I was due, about 3 weeks before you actually weren’t allowed to leave your house, I was in lockdown, he said ‘I don’t want you going anywhere’ ”<br>• Sweet 2021 “We did a lot of ordering online, but the things that I couldn’t order online I’d go like at stupid times of the week, like at 7am on a Sunday morning”<br>• Sweet 2021 “but it surprises me that ... you have to tell your own aunty, nanna, cousin to do or not do something and feel awkward about like the social relationships with them, whereas you’re just doing your job looking after your baby”<br>• Zinga 2022 “… I go in there and I have this list in my head and then I see all these people and I just think “Geez, okay. So what did I need to get again?”…And then I ended up spending money on crap that I don’t even need and I miss out on buying things that I actually did need because I’m a bit flustered…“<br>• Zinga 2022 “It’s really restricted me to where I can go…I would normally go to a bigger supermarket that has, I suppose, a bigger range of things, stuff like meats that are – and a bulk pack of, say, beef mince at a much cheaper price point than I can get around here…I have to say that meat is something I just haven’t been able to afford and have access to”
